# Characterization of SiO_2_ Etching Profiles in Pulse-Modulated Capacitively Coupled Plasmas

**DOI:** 10.3390/ma14175036

**Published:** 2021-09-03

**Authors:** Chulhee Cho, Kwangho You, Sijun Kim, Youngseok Lee, Jangjae Lee, Shinjae You

**Affiliations:** 1 Department of Physics, Chungnam National University, 99 Daehak-ro, Daejeon 34134, Korea; paulati@naver.com (C.C.); nemam2g@gmail.com (K.Y.); ys.dunphy@gmail.com (Y.L.); leejj3800@naver.com (J.L.); 2 Nanotech, Yongin 28431, Korea; kim_sijun@naver.com; 3 Institute of Quantum System (IQS), Chungnam National University, Daejeon 34134, Korea

**Keywords:** silicon dioxide etching, liquid fluorocarbon precursor, global warming potential (GWP), etch selectivity, capacitively coupled plasma, pulse-modulated plasma

## Abstract

Although pulse-modulated plasma has overcome various problems encountered during the development of the high aspect ratio contact hole etching process, there is still a lack of understanding in terms of precisely how the pulse-modulated plasma solves the issues. In this research, to gain insight into previously observed phenomena, SiO_2_ etching characteristics were investigated under various pulsed plasma conditions and analyzed through plasma diagnostics. Specifically, the disappearance of micro-trenching from the use of pulse-modulated plasma is analyzed via self-bias, and the phenomenon that as power off-time increases, the sidewall angle increases is interpreted via radical species density and self-bias. Further, the change from etching to deposition with decreased peak power during processing is understood via self-bias and electron density. It is expected that this research will provide an informative window for the optimization of SiO_2_ etching and for basic processing databases including plasma diagnosis for advanced plasma processing simulators.

## 1. Introduction

As the feature size of semiconductors continues to decrease, and the structure of semiconductors is altered from two to three dimensions, high-aspect-ratio contact (HARC) hole etching has emerged as one of the most important goals in the semiconductor manufacturing process. Achieving high-performance HARC hole etching requires high selectivity [1,2,3,4,5,6,7], anisotropic etching [8,9,10,11,12], and reduced charge damage [13,14,15,16]. As continuous wave (CW) plasma reacts with a consistent ion flux on a wafer surface, it is difficult to attain these aspects with CW plasma. Many researchers subsequently found that pulse-modulated plasma can meet the above requirements [17,18,19,20,21,22]; despite such findings though, an understanding of the effects of pulse-modulated plasma, namely the detailed plasma parameters and how they influence surface reactions on the wafer, has remained unclear.

To clarify the pulse-modulated plasma effects, it is necessary to diagnose the plasma and analyze the key plasma parameters. Etching is dominantly determined by certain key parameters, i.e., radical species density, ion energy, and electron density [23]. Thus, many studies have been conducted to understand the relationship between pulse-modulated plasma (hereafter, pulse plasma) parameters and the SiO_2_ etching characteristics. Samukawa et al. investigated SiO_2_ etching characteristics through electron density and self-bias in pulse-time modulated electron cyclotron resonance plasma [24]. Jeon et al. researched SiO_2_ HARC profiles in dual-frequency-pulsed capacitively coupled plasma (CCP) via XPS surface analysis and electron temperature [25]. Song and Kushner studied SiO_2_ etch rates and profiles through average radical fluxes and the electron energy distribution function (EEDF) in pulse-modulated dual frequency CCP simulation [26]. Tokashiki et al. studied SiO_2_ and poly-Si etch rates and profiles in pulse-modulated inductively coupled plasma through electron temperature, electron density, plasma potential, and ion density [27]. However, as Samukawa et al. and Tokashiki et al. did not consider radical density, the chemical etching characteristics could not be fully explained, and otherwise, plasma simulation research should be matched with experimental data. It is important to note that plasma etching is not simply determined by one or two plasma variables, but rather occurs as a complex interaction between numerous variables at different levels of influence. Therefore, the combination of the key plasma parameters should be considered to deepen the understanding of the relationship between plasma and etch profiles.

In this paper, SiO_2_ wafers were etched by pulse plasma while changing various external parameters to know the complex interactions between the plasma parameters, namely electron density, radical density, and self-bias. Using these plasma parameters, etch rates and selectivity were analyzed. Moreover, undesired etch profiles such as tapered etching and micro-trenching were analyzed through these variables.

## 2. Experimental Details

**Figure 1:**
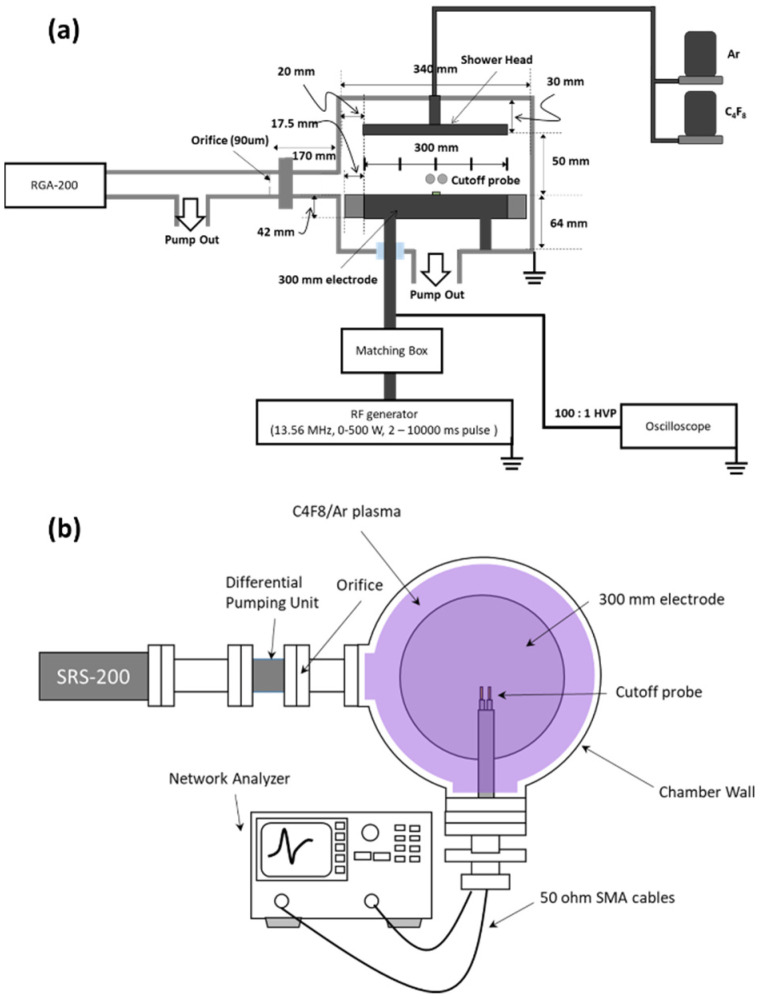
(**a**) Schematic of the chamber, and (**b**) diagnostic equipment set-up.

The experiments were performed using a radio frequency (RF) CCP source. A schematic outline of the CCP chamber is shown in Figure 1a. Discharge was maintained between 300-mm-diameter parallel-plate electrodes separated by 50 mm. The system was equipped with a 13.56 MHz RF frequency power generator connected to the bottom electrode via coaxial cable. The RF power generator RF5S (Advanced Energy, Denver, CO, USA) controlled the frequency and duty cycle of the pulse modulation power by independently varying power on/off time. The 1000 nm thick SiO_2_ was deposited on the silicon wafers by high-density plasma chemical vaporized deposition, and it was masked with a 200 nm poly-silicon hole patterned layer, with 350 nm diameter holes used. The ratios of the gap between the holes and the hole size were 1:1, 1.2:1, 2:1 and 5:1. This wafer was cut into coupon sizes and processed while placed in the center of the bottom electrode.

The diagnostics equipment set-up is shown in Figure 1b. The partial pressure of the radicals in the plasma was measured with an RGA-200 quadrupole mass spectrometer (QMS) (Stanford Research Systems, Sunnyvale, CA, USA). It was separated from the vacuum chamber by an orifice with a differential pumping unit system to create a vacuum in the QMS. The base pressure of the QMS chamber was under 10^−8^ Torr. To obtain radical density in plasma, the QMS signal should be converted. Singh et al. suggested the radical density converted method using appearance potential mass spectrometry (APMS) as follows [28]:

(1) $$\frac{n_{X}^{on}}{n_{Ar}^{off}}=\left(\frac{A_{X\to X^{+}}}{A_{Ar\to Ar^{+}}}\right)\left(\frac{\lambda_{Ar\to Ar^{+}}}{\lambda_{X\to X^{+}}}\right)\left[\frac{t\left(m_{Ar^{+}}\right)\theta \left(m_{Ar^{+}}\right)}{t\left(m_{X^{+}}\right)\theta \left(m_{X^{+}}\right)}\right]$$

In this equation, $n_{X}^{on}$ is *X* radical density, $n_{Ar}^{off}$ is *Ar* density in the chamber without plasma, *A* and λ are the slopes of the linear fits of the QMS signal and the cross-section to eliminate electron energy dependence, t(m) is the transmission efficiency of the quadrupole mass filter, and θ(m) is the detection coefficient of the detector. $n_{Ar}^{off}$ is obtained using the ideal gas law,

(2) $$p=n_{Ar}^{off}kT$$

where *p* is chamber pressure, *k* is the Boltzmann constant, and *T* is gas temperature. While this method is typically used to obtain absolute radical density, the APMS method requires a high-performance QMS in order to precisely control electron energy. In the current paper, to measure the absolute radical densities in the plasma, the APMS equation was changed [28] based on the quality of the QMS. A variation of this equation was used as follows:

(3) $$\frac{n_{X}^{on}}{n_{Ar}^{off}}=\left(\frac{S_{X\to X^{+}}}{S_{Ar\to Ar^{+}}}\right)\left(\frac{\sigma_{Ar\to Ar^{+}}}{\sigma_{X\to X^{+}}}\right)\left[\frac{t\left(m_{Ar^{+}}\right)\theta \left(m_{Ar^{+}}\right)}{t\left(m_{X^{+}}\right)\theta \left(m_{X^{+}}\right)}\right]$$

Here, *S* is the QMS signal, and σ is the ionization cross-section. As the QMS signals were obtained at 70 eV electron energy, 70 eV ionization cross-sections were used. The Ar reference data for this method was measured at a 20 mTorr chamber pressure.

Table 1 shows the parameters and symbols in Equations (1)–(3). The radical density was determined by averaging the measurement five times.

Here, Radical species C, CF, CF_2_, …, etc., were measured, and they were substituted as in Table 2. When the electron energy is set at 70 eV, the dissociative ionization of C_4_F_8_ in the QMS generate the gross error of radical density due to the high electron energy. To investigate the dissociative ionization effect on the gross error, we measured electron energy distribution in the QMS ionizer via an electric probe [29,30]. By sweeping the probe bias voltage from −12 to +80 V, the electron current was measured and the second derivative of the current–voltage curve, which is proportional to the electron energy distribution, was evaluated. As a result, it is found that the electrons mostly existed in the energy range of 0~15 eV, or 30~50 eV and there are two depletion regions between the two groups and over the electron energy of 50 eV when with 75 eV setting. The first depletion region in 15~30 eV energy is believed to be made by the ionization process of molecular gases in ionizer, more ionization creates a higher depletion of electrons. The second depletion region over 50 eV is believed to be made by the escape of electrons which can overcome the potential barrier of the ionizer. Therefore, ionization mostly occurs in the first depletion region. Moreover, in the cross-section of dissociative ionization, and direct ionization above the first depletion electron energy region, the direct ionization is higher than dissociative ionization [31]. Therefore, the estimated radical density is highly affected by direct ionization.

The electron density of the plasma was measured via a cutoff probe, which measures electron density with a simple method using microwaves [32]. The electron density was measured by averaging 50 times to get accuracy. Self-bias was measured with a high-voltage probe connected to an oscilloscope (Tektronix, Beaverton, OR, USA). The 100:1 high-voltage probe was connected to the bottom electrode. The voltage waveform is obtained using the high-voltage probe. Self-bias is the DC offset of this waveform. Etch profiles were obtained with a scanning electron microscope (SEM) (Topcon, Tokyo, Japan). The coupon wafer was cut into cross-sections and images were obtained through SEM. Since the depth of the etched hole is visible in the cross-sectional images, the etch rate can be obtained by dividing the depth of the hole by the processing time. The poly-Si mask etch depth is obtained by subtracting poly-Si height after the etching from 200 nm, which is the thickness height of the mask before the etching. As the SiO_2_ etch rate, mask etch rate is obtained by dividing mask etch depth to process time. The SiO_2_–mask selectivity was calculated by dividing the SiO_2_ etch rate by the mask etch rate.

**Figure 2:**
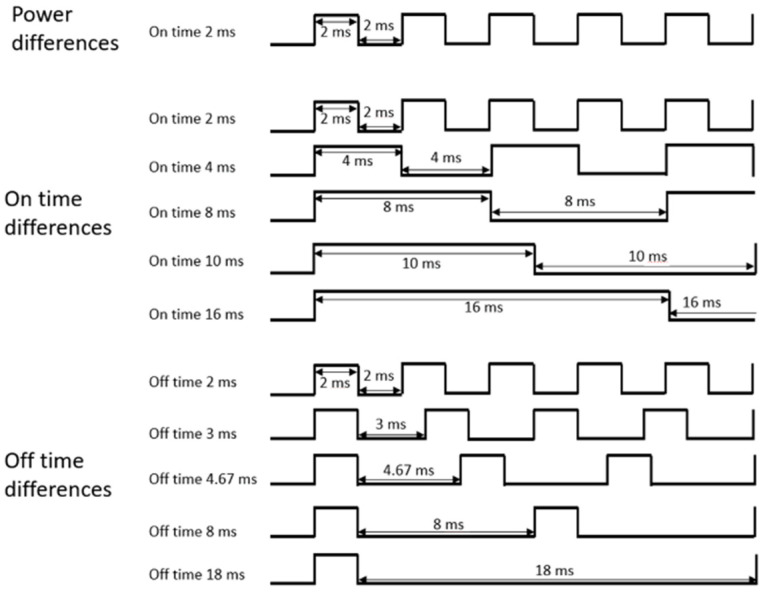
Pulse-modulated power scheme.

In this paper, etch profiles were obtained by varying three external parameters, namely power, pulse on-time, and pulse off-time, where the pulse change is explained through pulse on/off time, not frequency and duty cycle. To analyze the effect on pulsed plasma by directly controlling the discharge on time and discharge off time of the plasma, rather than the frequency and duty cycle of the plasma, the experiment was conducted by controlling the RF power on/off time. The on/off control scheme is shown in Figure 2.

In the first experiment, the pulse-modulated RF power applied to discharge the plasma was varied from 100 to 400 W, while the other parameters were kept constant. In the second experiment, the plasma pulse on- and off-time were varied together while the power was kept constant. In the third experiment, only the pulse off-time was changed while the other parameters remained constant. In all experiments, the benchmarked conditions, which have high SiO_2_ etch rate and selectivity, were as follows: 400 W power, 2 ms on-time, 2 ms off-time, 1:1 C_4_F_8_/Ar ratio, and 20 mTorr pressure. The total on-time in all experiments was set to 10 min, and accordingly, the processing time was changed for each experimental condition.

## 3. Results

**Figure 3:**
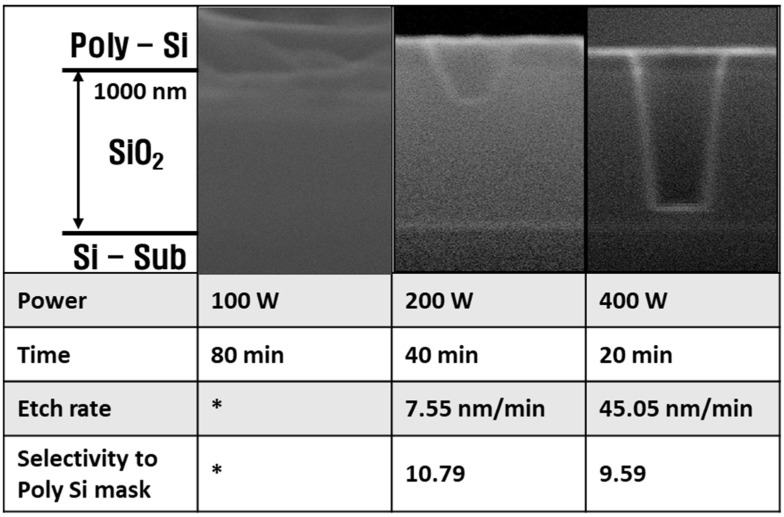
SiO_2_ etching profile, etch rate, and selectivity of SiO_2_/Si at increasing power. * Etch rate cannot be expressed.

Figure 3 shows the etch profiles, SiO_2_ etch rates, and SiO_2_ to Si selectivity with increasing pulsed RF power. Deposition occurs in the 100 W condition. Etch rate cannot be expressed here, so * is used. In order to equalize the sum of the total power in the etching process, experiments were performed by increasing the processing time as the power decreased.

At high power, the etch rate and selectivity appeared to be similar to other research [1,24], and anisotropic etching occurred. However, as the power decreased, the etch rate decreased, the selectivity increased, and the etch profile became isotropic. At low RF power, deposition occurred, despite the etching process.

**Figure 4:**
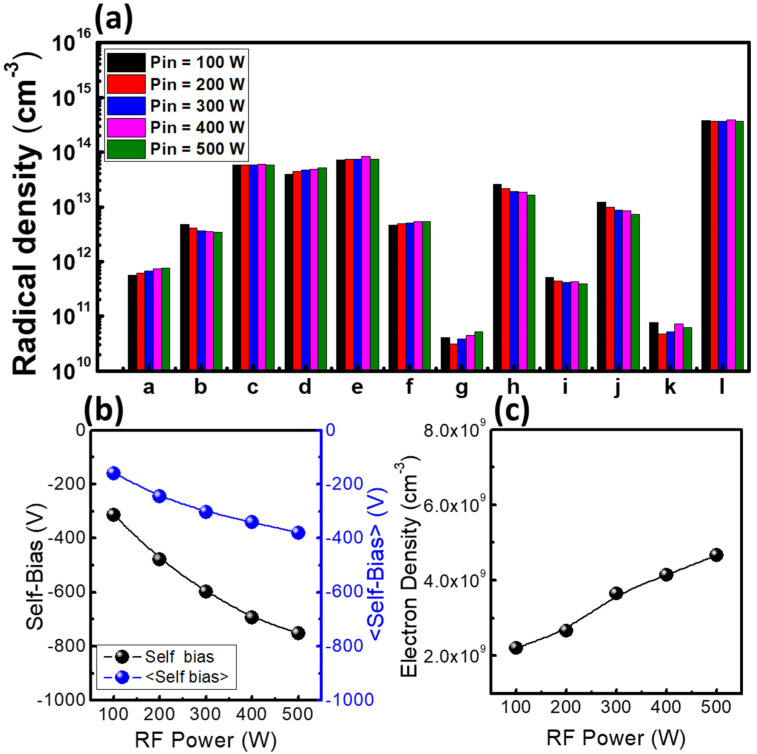
Diagnostic data as a function of input power: (**a**) radical densities, (**b**) self-bias and averaged self-bias, and (**c**) electron density. The parameters for these experiments were a CW RF power, a pressure of 20 mTorr, and a C_4_F_8_/Ar gas ratio of 1:1.

These etching characteristics were analyzed by plasma diagnostic data. Figure 4 shows the plasma diagnostic data for radical densities, the absolute value of self-bias, and the electron density as functions of RF power. As the RF power increased, the electron density and absolute self-bias, which can represent the ion bombardment energy, also increased. In addition, it was confirmed that chemical reactions in the plasma were increased by the increase in electron density, so that the density of the high mass molecules decreased while that of the low mass molecules increased. As most of the low mass molecules have a higher fluorine/carbon ratio (F/C ratio) than the high mass molecules, the low mass molecules are expected to deposit fewer polymer layers than the high mass molecules. With these results, the etch characteristics shown in Figure 3 can be analyzed. The ion bombardment energy is high at high power, so that the polymer layers deposited by C_x_F_y_ radicals can be sufficiently removed, in addition to the high density of the low mass molecules decreasing the formation of the polymer layers. On the other hand, at low RF power, the ion bombardment energy becomes insufficient to remove C_x_F_y_ polymers. Additionally, the increase in polymers with low F/C ratios such as C_3_F_4_ and C_3_F_5_ can contribute to the formation of the polymer layers. Therefore, the process changes from deposition to etching with increasing RF power.

**Figure 5:**
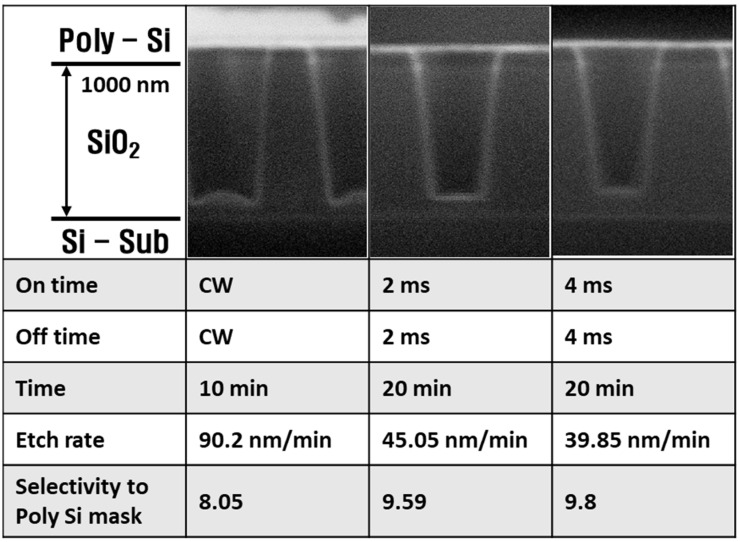
SiO_2_ etching profile, etch rate, and selectivity of SiO_2_/Si at increasing on/off times.

In Figure 5, the etch profiles, SiO_2_ etch rates, and SiO_2_ to Si selectivity are shown as functions of pulse on/off times, which were set to CW, 2 ms, and 4 ms. For an equal total power in all experiments, the etch time increased from 10 to 20 min when the power changed from CW to pulsed. In the CW mode, the micro-trench phenomenon occurred, but it was absent in the pulse plasma mode. When the power changed from CW to pulsed, the etch rate decreased from 90.2 to 39.85 nm/min, and the selectivity increased from 8.05 to 9.8.

**Figure 6:**
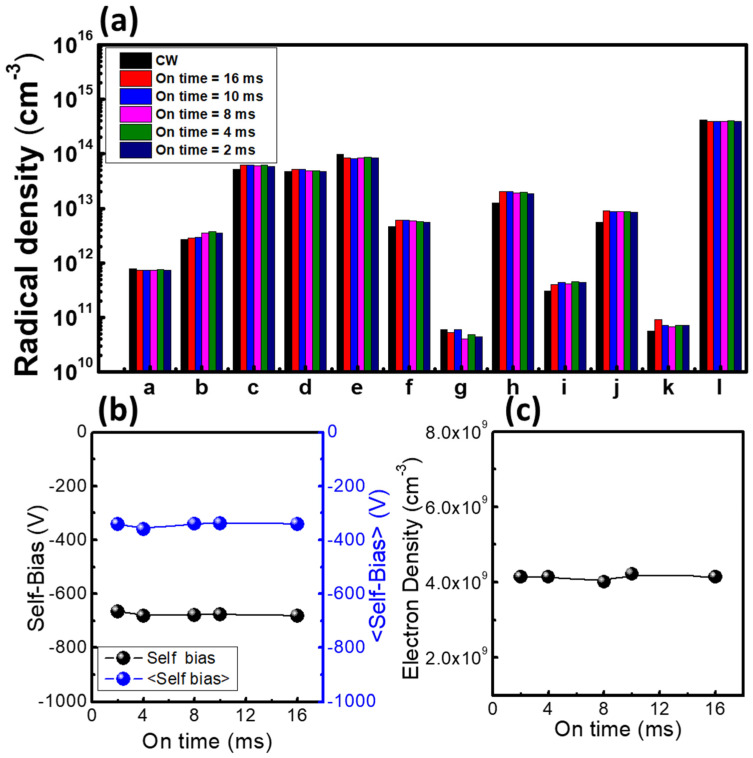
Diagnostic data as a function of on/off time: (**a**) radical densities, (**b**) self-bias and averaged self-bias, and (**c**) electron density. The parameters for these experiments were a RF power of 400 W, a pressure of 20 mTorr, a C_4_F_8_/Ar gas ratio of 1:1, and a pulse off-time equal to the on-time.

Figure 6 shows the radical densities, self-bias, and electron density as functions of the pulse on/off time, which ranged from 2 to 16 ms. The radical densities, self-bias, and electron density did not obviously change when the pulse on-time increased. Referring to Figure 5, the etch rate of the pulse plasma was half that of the CW plasma. The cause of the different etch characteristics between CW plasma and pulse plasma was that the pulse plasma had half the pulse on-time within the same period, meaning that the ion bombardment time of the pulse plasma was half that of the CW plasma. It should be noted that ions may not exist during the off-time considering their short lifetimes, while radicals can exist during the off-time due to their long lifetimes [18]; hence, radicals formed passivation layers on the SiO_2_ and poly-Si surface during off-time. As the passivation layers protecting the wafer were the same with both plasma types, the reduced ion bombardment with the pulse plasma resulted in decreased surface etching compared to CW plasma. Thus, pulse-modulated plasma had higher selectivity than CW plasma.

**Figure 7:**
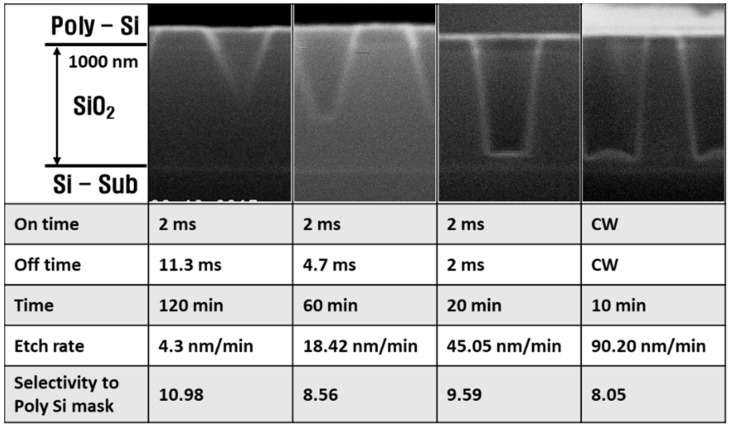
SiO_2_ etching profile, etch rate, and selectivity of SiO_2_/Si at decreasing off-times.

In Figure 7, the etch profiles, SiO_2_ etch rates, and SiO_2_ to Si selectivity are shown as functions of pulse off-time, which varied from 2 to 11.3 ms. The bottom of the etched feature was narrower than the top feature at the 11.3 ms off-time, giving a triangular etch profile. As the off-time decreased, the bottom feature width increased, leading to anisotropic etching. Additionally, the etch rate increased from 4.3 to 45.05 nm/min, and the selectivity decreased from 10.98 to 9.59.

**Figure 8:**
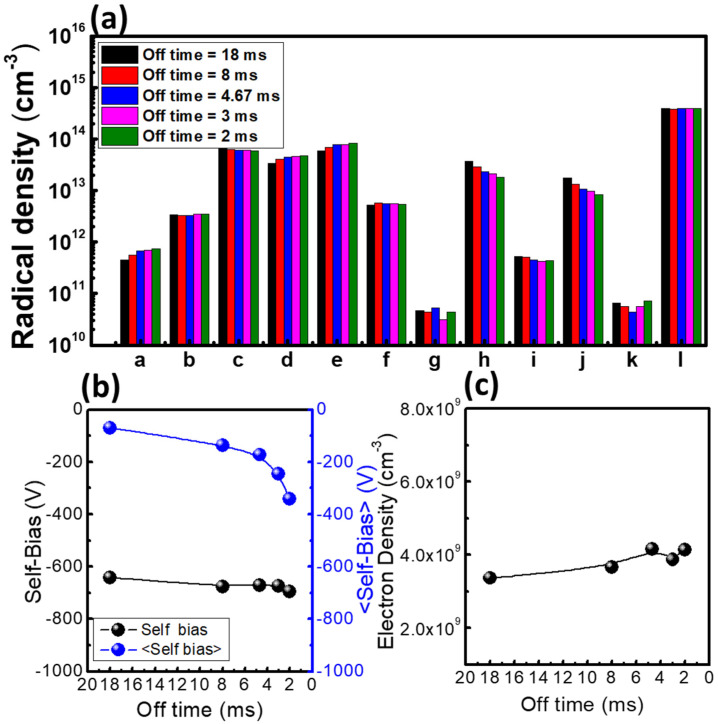
Diagnostic data as a function of off-time: (**a**) radical densities, (**b**) self-bias and averaged self-bias, and (**c**) electron density. The parameters for this experiment were a RF power of 400 W, a pressure of 20 mTorr, a C_4_F_8_/Ar gas ratio of 1:1, and a pulse on-time of 2 ms.

In Figure 8, the radical densities, electron density, and self-bias are shown with an off-time ranging from 2 to 18 ms. Here, the on-time was fixed to 2 ms. The C, F, CF_2_, and CF_3_ radical densities increased, while the C_2_F_4_ and C_3_F_5_ radical densities decreased with decreasing off-time. The cause of these results was that the low mass radicals combined to form high mass radicals during the plasma power off-time. These high mass products enhanced the passivation layer formation. The self-bias did not change much when the off-time decreased, while the time-averaged self-bias increased rapidly as the off-time decreased. The self-bias is obtained as the V_dc_ of the on-time voltage wave form; on the other hand, the averaged self-bias is obtained as the V_dc_ of the total voltage wave form. Therefore, a decreasing averaged self-bias does not lead to decreasing ion bombardment energy. These results show that the energy of the sputtered ions was the same, but as the sputtering time increased with decreasing off-time, the etch rate increased and the selectivity decreased. In the case of electron density, it affected the ion flux on the wafer surface. The electron density increased with the decreasing off-time, representing an ion flux increase. Therefore, both the ion sputtering rate and the chemical etching of the radicals increased as the off-time decreased.

**Figure 9:**
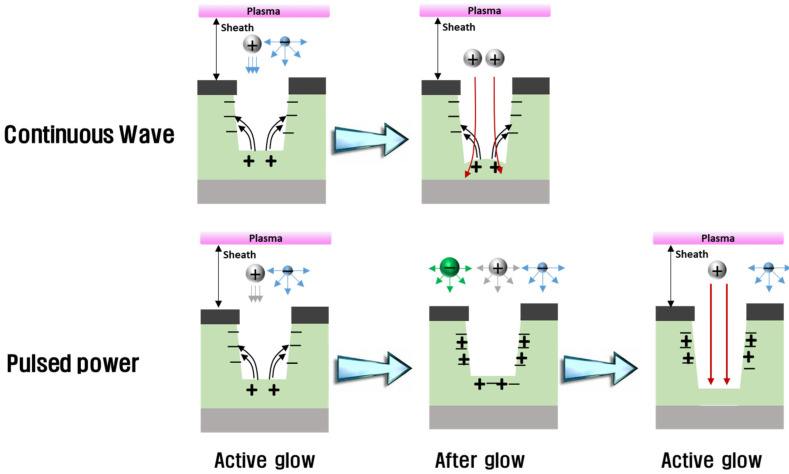
Micro-trench etching mechanism with continuous wave plasma and pulse-modulated plasma.

In the plasma process, not only are the etch rate and the selectivity important but so is the shape of the trench. As can be seen in the top-left panel of Figure 5, the edge of the bottom feature was etched lower than the center of the bottom feature in the CW condition. This phenomenon is called micro-trenching [33], and its cause has been reported [34]. It is also known that pulse-modulated plasma reduces the micro-trench effect [34]; Figure 9 represents this mechanism. Positive ions are accelerated in the plasma sheath, anisotropically toward the substrate, while electrons isotropically move in the plasma sheath. As a result, the bottom of the trench is positively charged because of the anisotropic positive ions, and the side of the trench is negatively charged from the isotropic electrons. Charge differences between the sides and bottom of the trench create an electric field, which bends the ions toward the side walls, resulting in micro-trenching. In the etch profiles from 2 and 4 ms pulse plasma in Figure 5, no micro-trenching appears. It has been reported that the ions losing directionality during the power off-time causes the disappearance of micro-trenching [34]; the time-varying self-bias data in the present work verified this.

**Figure 10:**
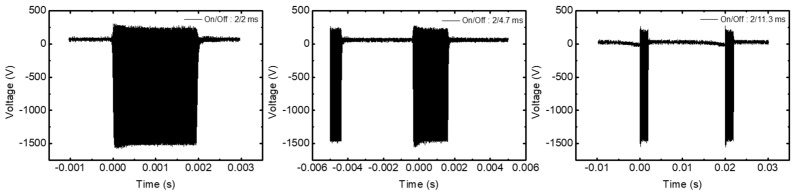
Input voltage of the powered electrode in pulse-modulated plasma at increasing off-time.

Figure 10 shows the time-varying input voltage of the powered electrode in pulse plasma as a function of off-time. The V_dc_ of the data is the self-bias. The ions are accelerated by the V_dc_; since V_dc_ is almost zero during pulse off-time, ions do not accelerate during the off-time. Therefore, the positive ions move isotropically like electrons, which reduces the micro-trenching phenomenon [34].

**Figure 11:**
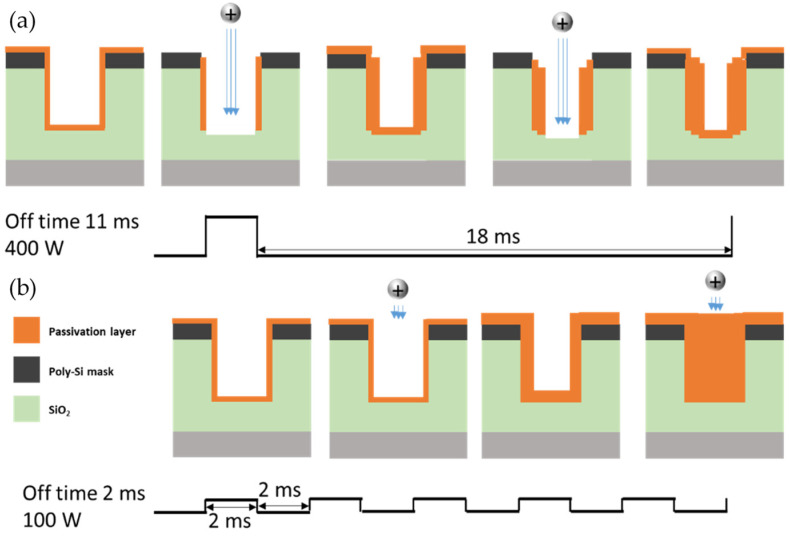
Etch profile mechanism in two different conditions: (**a**) RF power of 400 W and off-time of 11 ms giving a triangular etch profile, and (**b**) RF power of 100 W and off-time of 2 ms giving a deposition profile.

Referring to Figure 4 and Figure 8, the averaged self-bias from 100 W power with 2 ms off-time is −160 V, while that from 400 W power with 11 ms off-time is −120 V, showing that ion acceleration is lower at 400 W. Therefore, this condition should produce less etching than the 100 W condition. However, Figure 4 also shows that the 400 W condition is more deeply etched because the self-bias value, which means the on-time self-bias only, is high at −675 V. The shape of the etch profile is not anisotropic, but a triangle. On the other hand, the 100 W condition shows deposition rather than etching. Figure 11 shows the mechanism of triangular etching and deposition. A passivation layer deposits on the entire wafer surface due to radicals. As the radicals survive during power off-time because of their long lifetime, the thickness of the passivation layer increases under the 11 ms off-time condition. During the on-time, ions remove the passivation layer with high ion bombardment energy. Since the accelerating ions move straight, though, the sides of the passivation layer are not sufficiently removed, and deposition is repeated during the next power off-time. As a result, the side walls gradually narrow to form an etched triangle. In the 100 W condition, the passivation layer is also formed, but as the off-time is short, the thickness of the layer is smaller. Then during the power on-time, the accelerating ions do not remove the passivation layer because the self-bias is weak at almost −200 V. Therefore, although the averaged self-bias is higher, when the self-bias is weak during on-time, etching does not occur, but rather deposition.

## 4. Conclusions

To interpret pulse-modulated plasma etching, a SiO_2_ wafer with a poly-Si patterned mask was etched under various plasma conditions while the key plasma parameters—radical species density, self-bias, and electron density—were measured. The chemical properties of the plasma were interpreted by the radical densities, while the physical properties of the plasma were interpreted by self-bias and electron density. Additionally, using all plasma parameters, the shapes of the etch profiles were investigated. Results showed that when the RF power increased, the SiO_2_ etch rate also increased because of the increasing self-bias. Additionally, as the power on/off time increased, the micro-trenching phenomenon disappeared because negative ions neutralized the wafer surface. When the power off-time increased, the etch rate decreased since the high mass radicals increased and the averaged self-bias decreased. It was also found that a triangular etch profile occurred due to the low V_dc_ during the power off-time and the high V_dc_ during the power on-time.

As a fundamental study to understand pulse plasma characteristics and etching properties, the results here can be examined to clarify how plasma parameters change in pulse-modulated plasma and to analyze SiO_2_ etching results using the parameters. This fundamental understanding will help to interpret more complex experimental plasma etching properties.

## Figures and Tables

**Table 1:** Symbols representing the parameters used in Equations (1)–(3).

Parameters	Symbols
Linear fitting of QMS signal	*A*
Linear fitting of ionization cross section	λ
X radical Density	*n_X_*
Transmission probability	*t*
Detection probability	θ
Chamber pressure	*p*
Boltzmann constant	*k*
Gas temperature	*T*
QMS signal	*S*
Ionization cross section	σ

**Table 2:** Labels to be displayed in Figures 4a, 6a and 8a substituting each radical species to labels.

Radical Species	Labels
C	a
F	b
CF	c
CF_2_	d
CF_3_	e
C_2_F_3_	f
CF_4_	g
C_2_F_4_	h
C_3_F_4_	i
C_3_F_5_	j
C_4_F_6_	k
Ar	l

## Data Availability

Not applicable.
